# Spatial and Temporal Variation in Heterophil-to-Lymphocyte Ratios of Nestling Passerine Birds: Comparison of Blue Tits and Great Tits

**DOI:** 10.1371/journal.pone.0074226

**Published:** 2013-09-16

**Authors:** Jerzy Banbura, Joanna Skwarska, Miroslawa Banbura, Michal Gladalski, Magdalena Holysz, Adam Kalinski, Marcin Markowski, Jaroslaw Wawrzyniak, Piotr Zielinski

**Affiliations:** 1 Department of Experimental Zoology and Evolutionary Biology, Faculty of Biology and Environmental Protection, University of Lodz, Lodz, Poland; 2 Museum of Natural History, Faculty of Biology and Environmental Protection, University of Lodz, Lodz, Poland; 3 Department of Teacher Training and Biological Diversity Studies, Faculty of Biology and Environmental Protection, University of Lodz, Lodz, Poland; 4 Department of Ecology and Vertebrate Zoology, Faculty of Biology and Environmental Protection, University of Lodz, Lodz, Poland; CNRS, Université de Bourgogne, France

## Abstract

Environmental factors affecting trophic conditions act as stressors on nestling altricial birds. Access of parental birds to a sufficient supply of food in a limited period of the nestling stage differ in time and space, depending on nesting habitat, prey density and weather conditions. Heterophil-to-lymphocyte ratio (H/L) is considered as a reliable indicator of prolonged stress reaction in birds. In this study we examine if variation in H/L shows consistent spatio-temporal patterns in nestlings of two parids, blue tit *Cyanistes caeruleus* and great tit *Parus major*. We found that blue tit nestlings had on average higher H/L than great tit nestlings, which corresponds with the ecological sensitivity of these species. In both species H/L was higher in a poor parkland habitat than in a high quality forest habitat. In nestling blue tits, higher H/L values occurred in years characterized by more extreme weather conditions and worse caterpillar availability. Such consistent patterns of variation in the H/L ratio of nestling blue tits and great tits suggest that, when age-dependent effects are controlled, the ratio can be used as an indicator of physiological stress that is generated by food-related stressors differing in space and time. In particular, elevated H/L ratios are indicative of human-induced changes in the structure of breeding habitats.

## Introduction

A degree of specialization in utilization of habitats during breeding and non-breeding stages of life is variable among animals, including birds [1]. Species that are not strictly associated with a very special habitat can settle in different habitats, varying in suitability and quality, which enforces individuals or breeding pairs to decide where to settle to maximize benefits and minimize costs in fitness [2]. In addition, ecological conditions in settlement habitats differ also among years, which may change profitability of settlement decisions. In the case of breeding habitats, settlement decisions are directly translated into offspring numbers and quality [2], [3]. Inter-habitat and inter-annual differences in clutch size and other components of reproductive success have long been found in population studies [4]–[10]. Such differences have also been revealed in our study system [11], [12]. Breeding habitat preferences seem to result from variation in availability of nesting sites and food in interaction with the risk of predation and parasite infestations [3], [5], [13]–[16].

Unfortunately, variation in habitat quality is notoriously difficult to assess in the field [1], [7], [10], [16]. Such an assessment is important for population ecology, but also for conservation biology, especially in association with human-induced changes in habitats. Because different hematological and physiological characteristics of birds have been found to consistently differ among habitats in relation to habitat properties [17]–[25], an idea has been developed that ecological physiology could support conservation by providing some tools for assessing habitat quality [26]–[29]. Both ecological physiology and conservation biology are especially interested in physiological stress reaction of organisms in response to detrimental factors (stressors) occurring in the environment at different spatio-temporal scales [30]–[34]. Stress reaction is adaptive because it adjusts physiological functions to compensate for potentially harmful disturbances of homeostasis [35], but obviously allocation of resources in stress response is traded-off with other functions, especially immunity, and, therefore, costly [36]–[38]. As a result, prolonged stress responses developed under the influence of chronic stressors lead to fitness costs; habitats that produce chronic stressors are considered as low-quality environments [28], [29], [33], [39].

As mediators in vertebrate stress response, glucocorticoid hormone concentration in blood provides direct metrics of stress [40]–[42]. Because handling of birds for taking a blood sample as well as a capture method may constitute a strong stressor in response to which glucocorticoid level changes very rapidly, alternative indicators of stress have also been analyzed, including heterophil-to-lymphocyte ratio (H/L ratio, hereafter) and stress proteins (see [42] for review). The H/L ratio turned out to be a reliable indicator of chronic stress that develops over a longer time [18], [41], [43]–[45]. In nestlings of different bird species, the H/L ratio is sensitive to different stressors, especially factors associated with food conditions [32], [46]–[49], [60].

Nestlings of hole-nesting altricial birds grow in nests that are relatively safe and well isolated from external factors, which is considered to be the reason for a relatively long time of their post-hatching development [50], [51]. Therefore, the outside environment influences nestlings mostly indirectly through their parents, especially through variable quality of parental care, including nest sanitation, brooding, and, first of all, food amount, quality and delivery frequency. In blue tits *Cyanistes caeruleus* and great tits *Parus major* the involvement in parental care differs between the sexes but participation of both parents in feeding nestlings is essential for nestling growth and survival [52]–[54]. Amounts of food, preferably caterpillars, and regularity of its delivery to nestlings depends on its abundance and spatial distribution, which may differ between habitats and years [5], [11], [52], [53], [55]. Human-induced changes in habitat structure and function, such as forest fragmentation and management, are known to negatively influence insect abundance and bird foraging [56]–[58], leading to physiological stress in birds [32], [59], [39]. Moreover, exposure to human visitors may disturb the regularity of feeding nestlings by parental birds [25], [60]. All this suggests that stress indicators of nestling tits may indeed be used as indicators of habitat quality and should display some predictable patterns of variation.

Blue tits and great tits belong to the commonest and most numerous passerines in the Western Palearctic [52]. Although they evolved as forest species, adapted to conditions of deciduous and mixed forests, in contrast to other species of parids, blue tits and great tits also regularly nest in various tree patches, even in city centers [52]. This suggests that they are more ecologically plastic than other parids. In spite of this general plasticity, great tits are more generalist than blue tits with respect to the food of the breeding period, with blue tits being more specialized in foraging on tree canopy caterpillars as key food of nestlings [52], . Being strictly dependent on leaf-eating caterpillars, blue tits are also likely to be more sensitive than great tits to human-induced changes in the structure of habitats [57], [58].

The main idea of this paper is that the environment subjects nestling tits to stress mostly through the food delivered by parental birds. Since trophic conditions differ among habitats and years, variation in physiological indicators of stress is expected to display a consistent pattern. Because blue tits are a little different from great tits in their degree of dependence on caterpillars, an inter-species difference in stress indices is also expected. General physical conditions, which constitute a background for nestling development, may also impact nestlings independently of food. Particularly, high temperature is a potential stressor that might induce elevated H/L ratios in nestlings and might differ between study sites.

Consequently, the aim of this study was to examine the following predictions concerning the H/L ratio variation in nestling blue tits and great tits:

- because of higher ecological sensitivity of blue tits in comparison with great tits, we expected that a corresponding difference should occur in chronic stress response, reflected in the H/L ratio,

- because trophic conditions for growing nestlings are better in the forest site than in the parkland site, we predicted that the H/L ratio of nestlings should be higher in the parkland,

- because food abundance and weather conditions during the nestling period tend to differ among years, we expected that the H/L ratio should also be variable between years,

- in addition, since the study sites differ in tree cover and the degree of insolation, we checked if maximal temperatures differ between the sites.

## Materials and Methods

### Ethics statement

This study involved the sampling of a small amount of blood of free-ranging bird nestlings, c. 10 µL per nestling, taken from the ulnar vain. The work was conducted according to Polish legal guidelines. All procedures of bird handling and blood sampling were approved by the Local Ethical Committee for Animal Experiments in Łódź (Permit Number: 1/ŁB 530/2011) and the Director of State Office for Environment Protection (Permit Number: DOP-OZGIZ.6401.03.41.2011.dł), the latter being given at the national level. At each location, the study was carried out with permission of relevant land managers: the Director of Łódź Botanic Garden, the Director of Łódź Zoological Garden and the Director of Łódź Forestry Administration Unit. The permit to band birds was given by the National Central Office for Bird Ringing, Museum and Institute of Zoology, Warsaw.

### Study area, food monitoring and weather

The study of variation in the H/L ratio of nestlings was carried out in 2005–2006 and 2010–2012 for blue tits and in 2010–2012 for great tits, as part of a long-term project (initiated in 1999) on the breeding ecology of nest-box populations of common hole-nesting birds in central Poland. The study system is localized around the city of Łódź and encompasses two 10 km-distant areas differing in habitat types: a parkland site with c. 200 nest-boxes and a woodland site with c. 300 nest-boxes. Wooden nest-boxes with removable front wall [61] were used.

The parkland site (51°45’N; 19°24’E) consists of two units, Łódź Botanic Garden (67 ha) and neighboring Łódź Zoological Garden (17 ha). Both the gardens belong to a large parkland area extending to the east and, partly, north of the study area, within the western part of the city of Łódź; the whole area is a remnant of an ancient deciduous forest dominated by hornbeam *Carpinus betulus* and oaks *Quercus robur* and *Quercus petrea*. The vegetation of the Zoological Garden descends from that forest, but it has been heavily fragmented by paths, buildings and fenced, deforested exposition spaces for animals. There are some small patches artificially wooded with Scots pines *Pinus silvestris*, birches *Betula* and some exotic tree species. Only some very limited fragments of the Botanic Garden consist of forest patches, mostly with birches as predominating trees. The vegetation of the most part of this garden has been formed artificially for the purpose of plant exposition, so that the tree cover is patchy with a large area of tree-free spaces. Tree patches are a mosaic of different deciduous and coniferous trees, in large part exotic species. Both gardens possess networks of pedestrian pathways and children playgrounds for visitors. They are very popular leisure time spaces for inhabitants of Łódź and tourists, and may be very crowded during spring and summer. South and south-east sides of the Botanic Garden border on highly urbanized area of the town with numerous blocks of flats.

The woodland study site (51°50’N; 19°29’E), the Łagiewniki Forest, is a rich deciduous forest of considerable size, c. 1250 ha, located N-E of Łódź [62]. Some fragments of the forest that were chopped down in the 1940 s have subsequently been reforested. The majority of the forest comes directly from the ancient deciduous woodland typical for the geographical area of central Europe. It represents different plant communities, mostly oak and hornbeam forests. In the c.120 ha nest-box supplied study area that is located in the central part of the forest, oaks are dominating tree species. The tree age structure is mature with all age-classes represented, and many trees as old as c. 200 years present. Some dead tree logs are usually maintained at places where they have grown.

In addition to the differences in the structure of the tree cover and human influence, the parkland and woodland study areas differ with respect to some other factors of ecological importance [11]. The Łagiewniki Forest has rich animal communities, including predatory birds and mammals. No predatory mammals regularly occur in the gardens, with one pair of the sparrowhawk *Accipiter nisus* breeding in this area in some years. A consistent feature of the study sites is that tree-crown-leaf-eating caterpillars are more numerous in the woodland than in the parkland [11]. The abundance of caterpillars was monitored using the frassfall-collecting method [11], [63].

Data on weather for Łódź, mean temperature and total rainfall for May, as the month when the nestling stage of the first clutch of tits occurs, were extracted from the TuTiempo.net climate data archive - http://www.tutiempo.net/en/Climate/LODZ/124650.htm. In addition, the number of rainy days was calculated from records of daily rainfall in the same archive. The weather in May was highly variable among the years of the study (Table 1). In 2012, we recorded the local temperature in May in both study sites using DS1921G Thermochron iButton loggers. The loggers were set up under randomly drawn nest-boxes; 12 loggers in the forest site and 8 in the parkland site. Temperature was recorded to the nearest 0.5°C every hour. Mean and maximum daily temperatures for each logger were used to calculate corresponding monthly means and those were treated as unit data points to compare mean and maximum temperatures between the study sites.

**Table 1:** Descriptive characteristics of May weather in Łódź in the study years.

Year	Mean temperature (°C)	Total rainfall (mm)	Days with rain
2005	13.5	68.84	13
2006	13.6	47.25	16
2010	12.5	158	23
2011	14	61.48	10
2012	15.3	19.31	7

### Field and laboratory procedures

During the breeding season, the nest-boxes were inspected once a week to record variables describing breeding: species, laying date, clutch size and so on. Because a frequency of genuine second broods is low, especially in blue tits, variable between years and difficult to discriminate from repeat broods, we decided to pool second and repeat broods into the late-brood category that was analyzed for comparison with first broods. Except this simple comparison of the H/L ratio between first and late broods, this study focused mostly on first brood nestlings.

On day 13–14 after hatching, the nestlings were individually banded, measured and sampled for blood, usually between 09∶00 and 14∶00 H. All nestlings in every brood were measured (wing length to the nearest 1 mm) and weighed (to the nearest 0.1 g), while only a random subsample of 3–5 nestlings blind-drawn out of same-age nestlings from every brood was designated for taking blood samples (c. 10 µL taken from the ulnar vain) that were used to prepare blood smears on individually numbered microscope slides. The smears were dried in the field and stained in the laboratory using a commercial Microscopy Hemacolor kit (Merck Chemicals). The number of heterophils and lymphocytes for every blood smear was counted to sum up to 100 cells, applying a microscope under 1000x magnification with oil immersion. All microscope counts of blood cells were conducted by the same person (JS), with independent counts of heterophils and lymphocytes being highly repeatable (r_i_ = 0.78±0.11 SE, F_9,20_ = 11.94, P<0.00001 in blue tits, and r_i_ = 0.79±0.10 SE, F_9,20_ = 12.17, P<0.00001 in great tits). The ratio of the number of heterophils to the number of limphocytes (H/L ratio) was then calculated. Because some smears were faulty or non-readable, we finally analyzed smears for 649 nestlings from 175 blue tit broods (including only 7 late broods) and 607 nestlings from 206 great tit broods (including 41 late broods).

### Statistical analysis

The individual H/L ratios were ln-transformed (ln (1+H/L)) to meet assumptions of analyses, but are presented in a non-transformed form in the figures and the text. Because the H/L ratios of nestlings from the same brood are not independent, the ln-transformed H/L ratios were analyzed using mixed linear models, with brood Id included as a random factor controlling for clustering; degrees of freedom were estimated by the Satterthwaite method [64]. Effects of different factors on the H/L ratio were modeled in an ANCOVA style by first fitting a model that included wing length as an age-controlling covariate and all factors of interest. We previously confirmed that wing length is a reliable and precise measure of age in average trophic conditions [48]. The covariate was removed from the model when non-significant or retained when significant to produce the final model presented in the paper [65]. The comparison of the H/L ratios between first-brood nestling blue tits and great tits did not include the wing-length covariate because of a distinct difference in body size between these species; species identity was the main factor in this comparison, with year and site providing only a background. The year and site factors were basic factors in within-species analyses.

Habitat-specific temperatures recorded with DS1921G Thermochron iButton loggers in 2012 were compared between the parkland site and the forest site using t-test, with average May temperatures for individual loggers being unit data points. Peak annual frassfall values during 2005–2012 were compared between the sites using paired t-test.

All analyses were performed using IBM SPSS 20 software [64].

## Results

**Figure 1:**
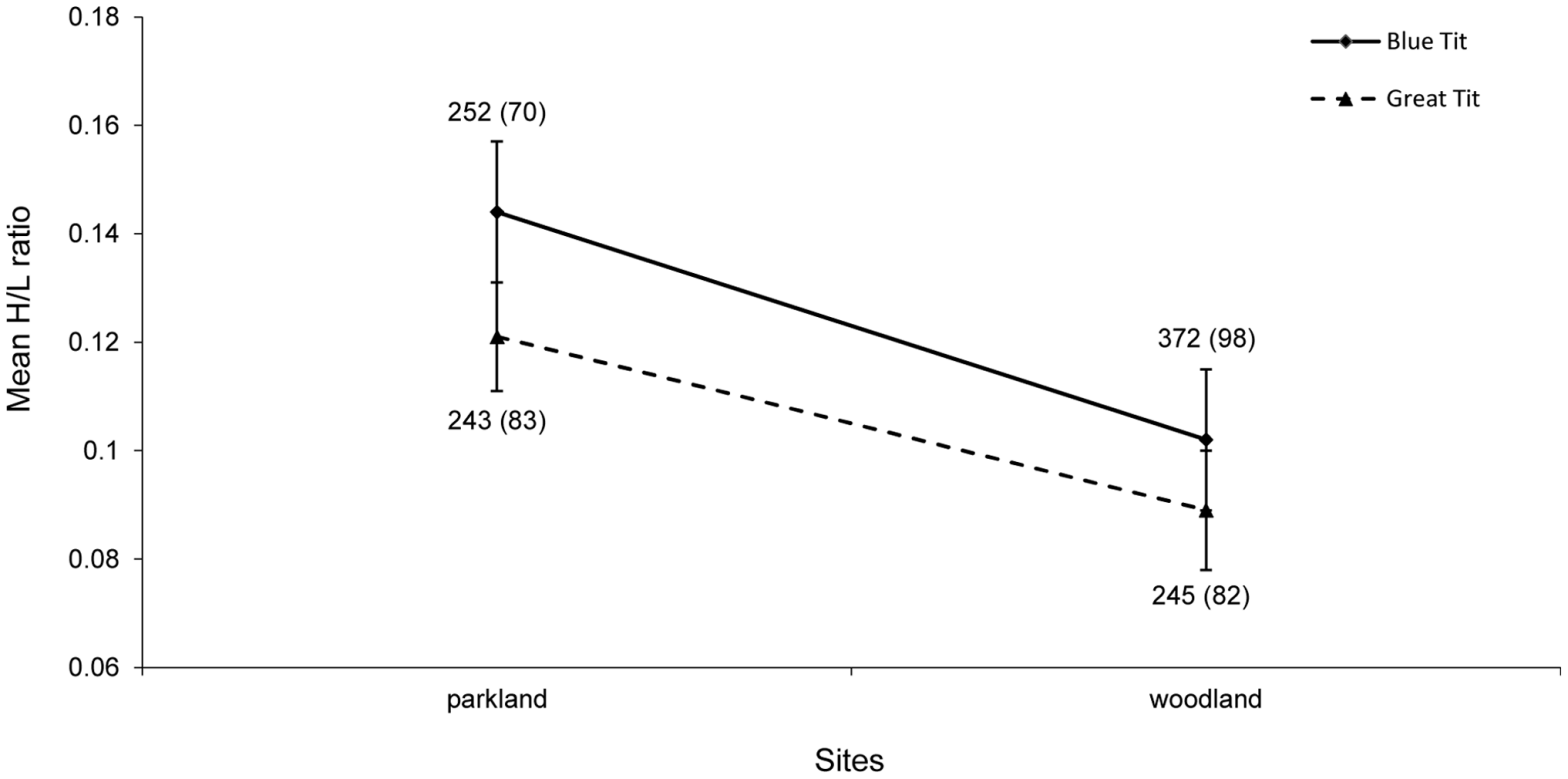
Comparison of blue tit and great tit nestlings in relation to inter-site variation in mean H/L ratios. Sample sizes are shown as the number of nestlings and, in parentheses, as the number of broods.

The analysis of inter-species variation in first brood at the background of the year and site factors showed that nestling blue tits had 16% higher mean H/L ratio than nestling great tits (mixed model ANOVA: F_1,384.599_ = 10.458, p = 0.001) (Fig. 1). In both species, significant intraclass correlations of H/L ratios were found (ICC = 0.89, Wald Z_1_ = 8.17, P<0.0001 for blue tits, and ICC = 0.72, Wald Z_1_ = 7.98, P<0.0001 for great tits, as calculated for the first clutch data).

**Figure 2:**
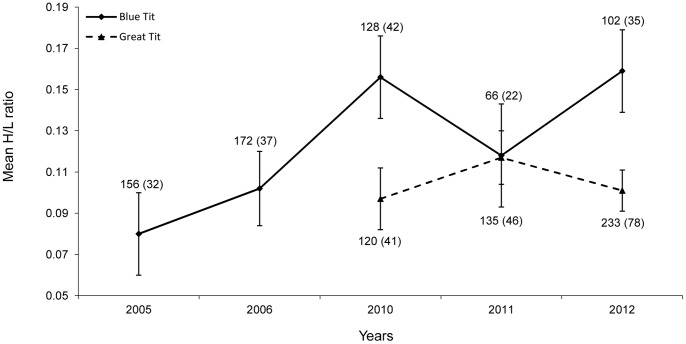
Comparison of blue tit and great tit nestlings in relation to inter-annual variation in mean H/L ratios. Sample sizes are shown as the number of nestlings and, in parentheses, as the number of broods.

In first brood nestling blue tits, the H/L ratio differed between sites and years (Table 2). Because there was no interaction between the site and year factors (Table 2), the main effects can be considered separately. The average H/L ratio was c. 30% higher in the parkland site than in the forest (Fig. 1). With respect to temporal variation, the lowest mean H/L value was recorded in 2005 and the highest values, c. twice as high as in 2005, were found in 2010 and 2012, with middle values found in 2006 and 2011 (Fig. 2). The Fisher LSD test showed that significant differences occurred between 2005 and 2010 (p = 0.007), 2005 and 2012 (p = 0.006) as well as between 2006 and 2010 (p = 0.05), and between 2006 and 2012 (p = 0.04), with all other pairwise differences being non-significant.

**Table 2:** Results of a mixed model ANCOVA for the H/L ratio of nestling blue tits, with wing length as covariate and year and site as factors.

Factor (covariate)	Df_n_	Df_d_	F	P
Year	4	175.81	4.047	0.004
Site	1	176.781	6.871	0.01
Year*site	4	176.688	1.869	0.118
(Wing length)	1	393.661	6.27	0.013

In first brood nestling great tits, the average H/L ratio was also notably higher, by c. 25%, in the parkland site than in the woodland site (Fig. 1, Table 3). Differences among the three years included, 2010–2012, were non-significant (Fig. 2, Table 3).

**Table 3:** Results of a mixed model ANOVA for the H/L ratio of nestling great tits, with year and site as factors.

Factor	Df_n_	Df_d_	F	P
Year	2	158.897	0.965	0.383
Site	1	158.924	5.412	0.021
Year*Site	2	158.897	0.05	0.951

For both tit species we also compared first broods with late broods at the background of the habitat and year factors. The average H/L ratio did not differ between the two categories of broods in either species (mixed model ANCOVA: F_1,184.721_ = 1.84, p = 0.18, with wing length as the significant covariant F_1,417.588_ = 6.588, p = 0.011 for blue tits, and mixed model ANOVA: F_1,213.409_ = 0.46, p = 0.50 for great tits).

The mean site-specific May 2012 temperature was c. 0.5°C higher in the parkland site than in the forest, the difference being statistically significant (15.06°C in the parkland v. 14.53°C in the forest; t_18_ = 3.99, P<0.001). A difference in mean maximum temperature was negligible and non-significant (19.70°C in the parkland v. 19.71°C in the forest; t_18_ = 0.07, P = 0.95). Average weather conditions in May were similar and relatively mild in 2005, 2006 and 2011, with mean monthly temperature ranging from 13.5°C to 14.0°C, and total rainfall between 47.25 mm and 68.84 mm. By contrast, May weather in 2010 and 2012 deviated from average conditions in two opposite directions. May 2010 was cold (12.5°C) and rainy (158 mm), whereas May 2012 was warm (15.3°C) and dry (19.31 mm).

**Figure 3:**
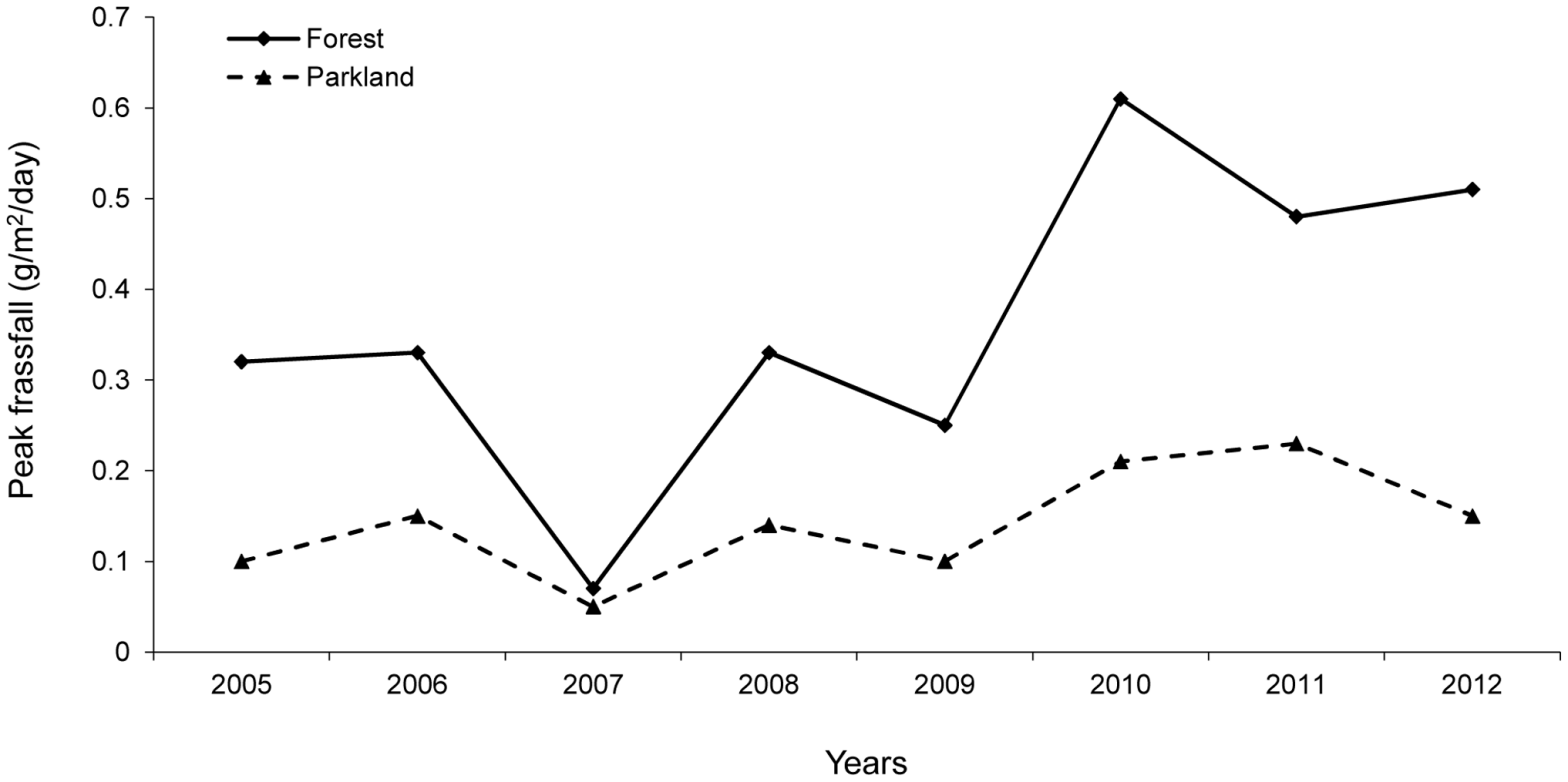
Comparison of the forest site and the parkland site with respect to inter-annual variation in the peak abundance of tree-canopy caterpillars, as measured by the peak amount of caterpillar frassfall.

The study sites notably differ in the abundance of caterpillars, as measured by peak frassfall (Fig. 3), with caterpillars in the forest being regularly more than twice as abundant as in the parkland (2005–2012 mean peak frassfall: 0.14 g/m^2^/day ±0.02SE in the parkland v. 0.36 g/m^2^/day ±0.06SE in the forest; paired t-test t_7_ = 5.23, P = 0.001).

## Discussion

Variation in the heterophil-to-lymphocyte ratio reported in this study revealed significant inter-species, inter-site and year-to-year differences. The H/L ratio in nestling blue tits was higher than in great tits. In both species, the H/L ratio was higher in the urban parkland site than in the forest site. In the blue tit, but not in the great tit, the ratio was significantly variable between years. In addition to these patterns, a notable part of the total variance in the H/L ratio lies between broods, 89% for blue tits and 72% in great tits, as shown for the first clutch by estimation of intraclass correlations [64], [66]. This suggests that nestlings from the same brood tend to be non-randomly similar to one another with respect to the individual H/L ratio.

Although the H/L ratio was shown to be a reliable indicator of different kinds of stress influencing birds [18], [41], [43], [44], some difficulties with interpretations are likely to arise. The H/L ratio is an index dependent on changes in two cell kinds engaged in immune response; heterophils are phagocytes involved in the first-line innate defense against infections, while lymphocytes are involved in the highly specific acquired defense [45], [67]. Since different cellular components of the immune system may develop along different trajectories with the growth of an organism, the age must be included in all comparisons in the case of studying the H/L ratio of nestlings [42], [68].

Moreover, even if comparisons concern nestlings of the same age, the H/L ratio and the level of circulating corticosterone as stress indicators respond to different types of environmental stressors, at a different rate and on different time scales [42], [49]. Glucocorticoids are biochemical mediators directly involved in stress reactions; their blood concentration may be elevated immediately after a stressor starts to influence an organism, whereas changes in leucocyte profiles leading to changes in the H/L ratio last longer [42]. Muller et al [49] found that in nestling Eurasian kestrels *Falco tinnunculus* the level of corticosterone responded with elevation to severe starvation and anthropogenic disturbances, while the H/L ratio increased in response to prolonged food-related, ectoparasitic, social and ecological stressors. Both these stress indicators responded to very severe stressors, potentially threatening to nestling survival. Because in studies on poultry it was found that intense stress may stimulate an increase in the number of heterophils and a decrease in the number of limphocytes, Maxwell [69] postulated that the H/L ratio is a good indicator of chronic mild to moderate stress.

Several experimental studies on birds included the nestling H/L ratio as one of response variables [32], [47], [48], [60], [70]–[72]. Some brood-size manipulations were reported not to affect the H/L ratio of nestlings [70]–[72], while the remaining ones produced significant effects, with nestlings in enlarged broods usually showing an elevated H/L ratio [32], [47], [60]. Brood-size manipulations change parental effort, amount and quality of food, feeding rate and social interactions between nest-mates; all these factors enhance stress in enlarged broods and may weaken stress in reduced broods [60]. The provision of extra-food makes rearing conditions easier, thus potentially reducing stress. We conducted previously such an experiment on great tits and found that nestlings in broods supplemented with additional food had a lower H/L ratio than nestlings in non-manipulated control broods [48]. In both types of experiments physiological stress seems to be directly or indirectly related to food and its distribution among nestlings.

Also ecological correlates of the H/L ratio suggest that food mediates in generating stress affecting nestlings. Hoi-Leitner et al. [46] found a negative correlation between granulocyte-to-lymphocyte ratios in nestling serins *Serinus serinus* and the abundance of food in breeding territories and nestling growth indices, which leads to the conclusion that better nutrition results in lower stress indicators. Moreno et al. [44] showed negative relations of stress indicators (the H/L ratio and stress proteins HSP60) with indicators of nestling nutrition. Suorsa et al. [32] revealed in nestling Eurasian treecreepers *Certhia familiaris* that broods in larger, less fragmented forest patches, where foraging is more efficient, were characterized by a lower H/L ratio. Because habitats and years tend to differ in the amounts and the availability of food in relation to specific abiotic and biotic conditions, it would be expected that indicators of stress should display corresponding patterns of spatio-temporal variation.

Accordingly, the patterns we report here seem reasonable. The higher average H/L ratio in nestling blue tits than in nestling great tits may suggest that the former are more sensitive to environmental stressors than the latter, which is consistent with the ecology of both these species. Although both species are commonly described as ecologically similar to each other, the blue tit is more than the great tit specialized in the preference for deciduous breeding habitats and in feeding nestlings with tree canopy caterpillars [52], [53], [73], [74]. In our study system, we previously found that the food of nestling great tits contained a lower percentage of caterpillars than in the case of blue tits and that the proportion of caterpillars in the diet was lower in the parkland than in the forest in great tits, whereas there was no difference in blue tits [82]. This suggests that in generally worse trophic conditions of the parkland site great tits preyed more frequently on alternative prey, while blue tits kept foraging on caterpillars, probably at some cost that might generate additional stress. In accordance with this, there seems to exist a slight tendency for the difference in the H/L ratio between nestling blue tits and great tits to be larger in the parkland site than in the forest site (Fig. 1). It seems probable that such subtle inter-specific differences in the H/L ratios are detectable only when both species are studied in the same sites and years.

Lower average H/L values found in the forest site than in the park site are likely to be caused by the availability of food. Caterpillars, the key food of nestling tits [52], [53], [55], [75], [76], are more than twice as abundant in the forest site than in the park site, as shown in this study and previous papers [11], [25]. This factor certainly makes rearing conditions more comfortable for parental birds and, therefore, for nestlings. In addition to the low abundance of caterpillars, the parkland site has also a very fragmented tree-cover and great numbers of human visitors who spend their leisure time there. Habitat fragmentation is known to impose difficulties on foraging birds [57], [58] and was shown to elevate stress in nestlings [32]. Human activity may interfere with feeding visits of parents at their nests and, thus, is likely to disturb the rate of nestling feeding.

Besides food, the inter-habitat difference in the H/L ratios could potentially result from some other habitat-specific effects. Because of tree-cover fragmentation, nest-boxes in our park habitat might be overheated as a result of more intense insolation than in the forest site. However, even in the very warm May 2012, mean maximum temperature did not differ between the sites, with mean monthly temperature being slightly higher in the parkland site than in the forest. This excludes a possibility of overheating as a major stressor.

Although inter-habitat differences in physiological parameters seem more interesting than inter-year differences, the latter are also a notable components of natural variation that need to be examined. Variation in the H/L ratio among years, in this study found in blue tits, but not in great tits, may also be associated with differences in food availability. The availability of caterpillars for foraging adult birds depends not only on the prey abundance but also on prevailing external conditions, with rainfall being a hindering factor in prey collecting [77]. In comparison with a very high density of caterpillars in 2003–2004 [11], their abundance was rather low in 2005–2006 and middle in 2010–2012. As a consequence of weather prevailing during the nestling stage of breeding, conditions for foraging and feeding nestlings were better in 2005–2006 and 2011 than in 2010 and 2012. The extreme weather during the nestling period may affect food availability and regularity of feeding visits, which would impact on nestlings as a stressor. This would be consistent with the H/L ratio variation among years in the blue tit. In the case of great tits, we did not study the H/L ratio in 2005 and 2006, and the values in 2010–2012 did not significantly differ among years. Although some spatial and temporal patterns were revealed in different physiological characteristics of nestling birds [21], [22], [25], [78], [79], we are not aware of any other study showing variation of the H/L ratio in relation to different habitats and years. Indication of the existence of some spatio-temporal variation in great tit nestlings may be inferred from the data shown by Norte et al. [80].

Though we expected that nestling H/L ratios should be lower in genuine first broods than in repeat and second broods (late broods), we did not find any difference. However, samples of late broods were small for both tit species, which reduced power of analyses. Timing of hatching did not affect the H/L ratio in another study on great tit nestlings [81], but it was significant in kestrels [49].

To sum up, consistent patterns of variation in the H/L ratio of nestling blue tits and great tits support the idea that the ratio can be used as an indicator of physiological stress that is generated by food-related stressors differing in space and time. The inter-species difference in the H/L ratio reflects a difference in ecological sensitivity between blue tits and great tits.
